# Fingolimod (FTY720-P) Does Not Stabilize the Blood–Brain Barrier under Inflammatory Conditions in an *in Vitro* Model

**DOI:** 10.3390/ijms161226177

**Published:** 2015-12-10

**Authors:** Michael K. Schuhmann, Stefan Bittner, Sven G. Meuth, Christoph Kleinschnitz, Felix Fluri

**Affiliations:** 1 Department of Neurology, University of Würzburg, Würzburg 97080, Germany; schuhmann_m@ukw.de (M.K.S.); kleinschni_c@ukw.de (C.K.); 2 Department of Neurology, University Medical Center of the Johannes Gutenberg-University Mainz, Mainz 55131, Germany; stefan.bittner@unimedizin-mainz.de; 3 Department of Neurology, University of Münster, Münster 48149, Germany; sven.meuth@ukmuenster.de; 4 Department of Physiology I-Neuropathophysiology, University of Münster, Münster 48149, Germany

**Keywords:** FTY720-P, blood-brain barrier, rat brain microvascular endothelial cell culture, inflammation, tight junctions

## Abstract

Breakdown of the blood-brain barrier (BBB) is an early hallmark of multiple sclerosis (MS), a progressive inflammatory disease of the central nervous system. Cell adhesion in the BBB is modulated by sphingosine-1-phosphate (S1P), a signaling protein, via S1P receptors (S1P_1_). Fingolimod phosphate (FTY720-P) a functional S1P_1_ antagonist has been shown to improve the relapse rate in relapsing-remitting MS by preventing the egress of lymphocytes from lymph nodes. However, its role in modulating BBB permeability—in particular, on the tight junction proteins occludin, claudin 5 and ZO-1—has not been well elucidated to date. In the present study, FTY720-P did not change the transendothelial electrical resistance in a rat brain microvascular endothelial cell (RBMEC) culture exposed to inflammatory conditions and thus did not decrease endothelial barrier permeability. In contrast, occludin was reduced in RBMEC culture after adding FTY720-P. Additionally, FTY720-P did not alter the amount of endothelial matrix metalloproteinase (MMP)-9 and MMP-2 in RBMEC cultures. Taken together, our observations support the assumption that S1P_1_ plays a dual role in vascular permeability, depending on its ligand. Thus, S1P_1_ provides a mechanistic basis for FTY720-P-associated disruption of endothelial barriers—such as the blood-retinal barrier—which might result in macular edema.

## 1. Introduction

Multiple sclerosis (MS) is a progressive inflammatory disease of the central nervous system (CNS) [1]. This disabling disease is characterized by multifocal demyelination, axonal loss, activation of glial cells, and infiltration by immune cells [1]. An early key event in the pathogenesis of MS is the loss of blood-brain barrier (BBB) integrity. Thereby, T helper lymphocytes secrete interleukin 17, which disrupts the BBB allowing efficient penetration of inflammatory cells into the brain [2]. Expression of matrix metalloproteinases (MMPs) further facilitates the migration of inflammatory cells [3].

The BBB consists of specialized brain endothelial cells which are supported in their barrier function by surrounding glial cells [4]. The function of the BBB is highly dependent on the expression and appropriate localization of tight junction (TJ) and adherens junction (AJ) complexes between the brain endothelial cells [5]. TJ proteins include claudins, occludin, junctional adhesion molecules (JAMs), and zonula occludens protein (ZO)-1 and ZO-2 [6]. The main function of occludin appears to be in TJ regulation [7]. In the BBB, expression of the proteins claudin 3, claudin 5, and possibly claudin 12 are considered to contribute to the high transendothelial electrical resistance (TEER) [8]. In MS lesions, abnormal occludin, ZO-1 and JAM-A expression correlated with active demyelination and BBB leakage [9,10].

Cell adhesion in the BBB is modulated by sphingosine-1-phosphate (S1P)—a signaling protein—via S1P receptors. In particular, endothelial S1P receptor-1 (S1P_1_) is involved in formation of intercellular adherens junctions and maintenance of BBB integrity [11]. Fingolimod (Gilenya™, FTY720-P)—a functional S1P_1_-antagonist—has been shown to improve the relapse rate in relapsing-remitting MS by preventing the egress of lymphocytes from the lymph nodes [12]. However, the role of FTY720-P in modulating TJ—in particular on occludin, claudin 5, and ZO-1—has not been elucidated so far. Furthermore, studies addressing the effect of FTY720-P on an *in vitro* BBB model are sparse and most often performed under non-inflammatory conditions [13,14,15]. This study aims to investigate whether FTY720-P (i) alters endothelial permeability in an *in vitro* BBB model under inflammatory conditions via S1P_1_ modulation; (ii) changes expression of MMP-2 and MMP-9; (iii) influences the amount of TJ proteins (occludin; claudin 5; ZO-1); and (iv) acts on the S1P/extracellular signal-regulated protein kinase (erk) 1 and 2 signaling pathway.

## 2. Results

### 2.1. FTY720-P Does Not Enhance Endothelial Barrier Function in Rat Brain Microvascular Endothelial Cell (RBMEC) Cultures

**Figure 1:**
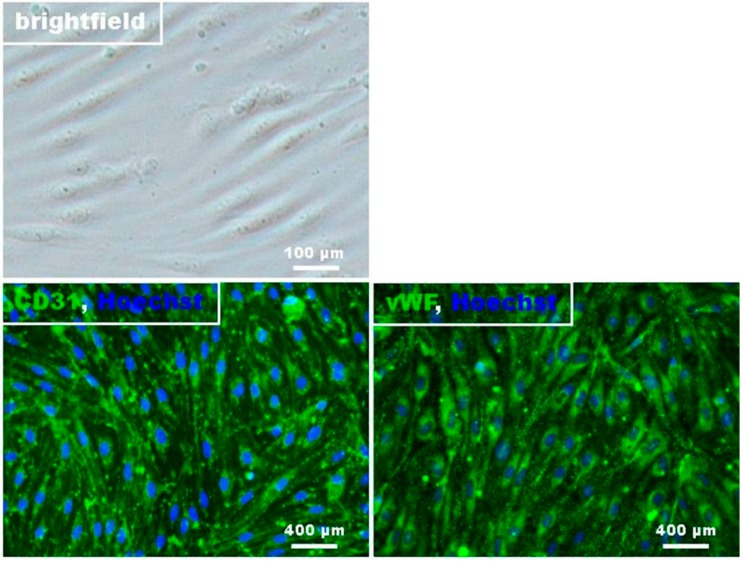

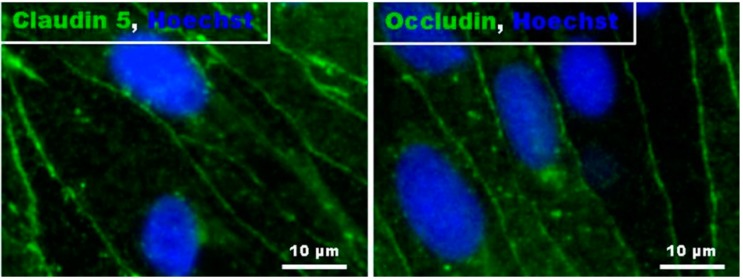
Histological characterization of rat brain endothelial cells. Phase contrast image of confluent RBMEC revealed that the cells express a spindle shaped morphology. Micrographs of immunofluorescence staining against Hoechst (blue), CD31, vWF, claudin-5, and occludin (green).

The main effect of FTY720-P in MS is the alteration of lymphocyte trafficking via modulation of S1P_1_ [16]. Brain microvascular endothelial cells (BMECs) represent a possible additional target for FTY720-P in MS patients by directly interfering with the function of the BBB, since BMECs also express S1P_1_ [17]. In a first set of experiments, the endothelial phenotypic morphology and the presence of a monolayer was confirmed by phase-contrast microscopy and immunostaining of the cells with the endothelial cell markers CD31 and von Willebrand factor (vWF) (Figure 1). About 95% of all RBMECs were vWF-positive indicating a high degree of cell culture purity. The barrier function of the present cell cultures was verified by the detection of the TJ-proteins claudin-5 and occludin.

First, the effect of FTY720-P on RBMECs under inflammatory conditions was examined. Five days after seeding cell cultures, RBMECs were exposed to an inflammatory milieu of interferon γ (IFNγ) and tumor necrosis factor α (TNFα) (100 IU each) and incubated with either a control medium (vehicle) or FTY720-P in three different doses (1, 10, and 100 nM) for 18 h. TEER was continuously measured during these 18 h, which enabled the integrity of the BBB to be determined *in vitro*. Incubation of RBMEC cultures with 550 nM hydrocortisone served as a positive control.

**Figure 2:**
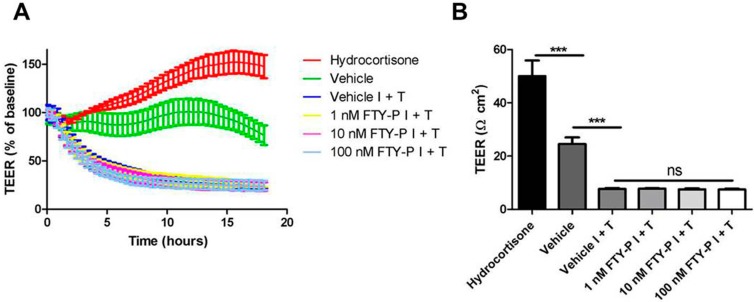
Transendothelial electrical resistance (TEER) of rat brain microvascular endothelial cell (RBMEC) cultures exposed to inflammatory conditions. (**A**) Time- and dose-dependent effect of FTY720-P (FTY-P; 1, 10 and 100 nM; *n* = 4) on TEER in RBMECs exposed to interferon γ and tumor necrosis factor α (I + T; 100 IU each) for 18 h compared with TEER of RBMECs in an inflammatory milieu alone (Vehicle I + T; *n* = 4) and under homeostatic conditions (Vehicle; *n* = 4). Hydrocortisone treatment (HC; 550 nM; *n* = 3) was used as a positive control; (**B**) Absolute TEER values of RBMECs 18 h after exposure to I + T (100 IU each) and FTY720-P treatment (*n* = 3 or 4). *** *p* < 0.001; ns, not significant.

TEER of vehicle-treated RBMECs decreased significantly after exposure to IFNγ and TNFα compared with RBMECs under homeostatic conditions (7.8 ± 0.3 Ω·cm^2^ *vs.* 24.6 ± 2.5 Ω·cm^2^; *p* < 0.001). Adding FTY720-P in three different doses to RBMECs exposed to an inflammatory milieu did not increase TEER compared with vehicle-treated RBMECs under the same conditions (Figure 2A,B).

**Figure 3:**
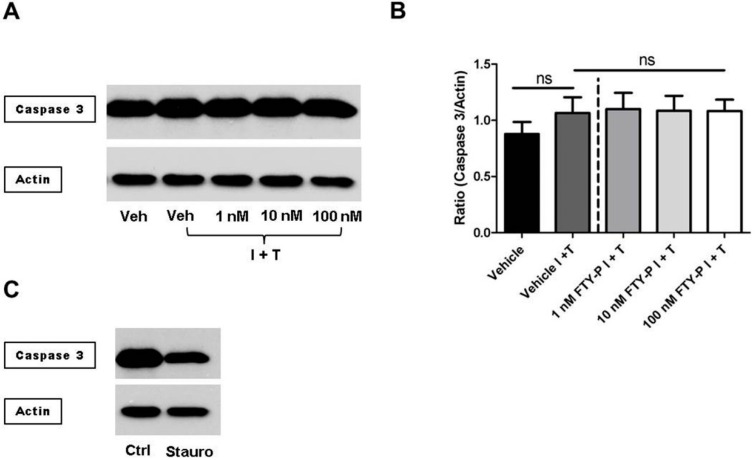
Analysis of apoptosis in rat brain microvascular endothelial cells (RBMECs) exposed to inflammatory conditions and incubated with FTY720-P. (**A**,**B**) Western blot analyses and densitometric quantification of the amount of full-length caspase 3 protein in RBMEC cultures after exposure to interferon γ and tumor necrosis factor α (I + T; 100 IU each) and treatment with FTY720-P (FTY-P; 1, 10 and 100 nM; *n* = 4) for 18 h compared with untreated cultures under inflammatory conditions (Vehicle I + T; *n* = 4) as well as with cultures under a homeostatic milieu (Vehicle; *n* = 4); (**C**) Staurosporine treatment (Stauro; 1 μM for 2 h) was used as a positive control. Ctrl, control; ns, not significant; Veh, vehicle.

Caspase-mediated apoptosis of cerebral endothelial cells might contribute to changes of their barrier function [18]. In this context, apoptosis might be due to high concentrations of IFNγ and TNFα or induced by a high (*i.e.*, 100 nM) concentration of FTY720-P. Therefore, a caspase 3-mediated apoptotic effect of these cytokines alone or in combination with FTY720-P on RBMECs was investigated using Western blot analysis followed by denistometric quantification. Neither the inflammatory conditions nor FTY720-P added to the inflammatory milieu induced caspase 3 activation or endothelial cell death (Figure 3A,B).

### 2.2. FTY720-P Does Not Alter the Amount of MMP-2 and MMP-9 Proteins in RBMEC Cultures

**Figure 4:**
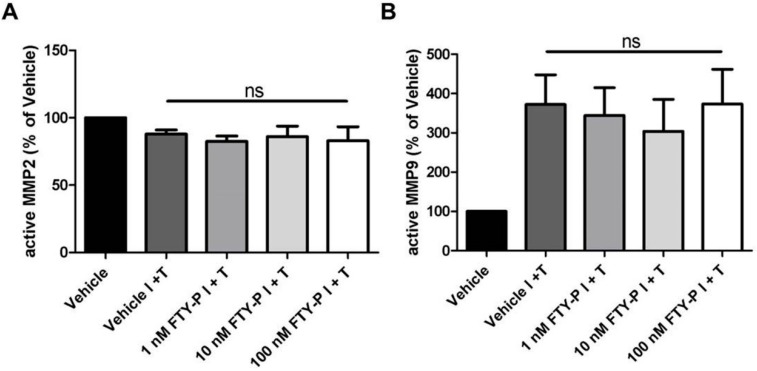

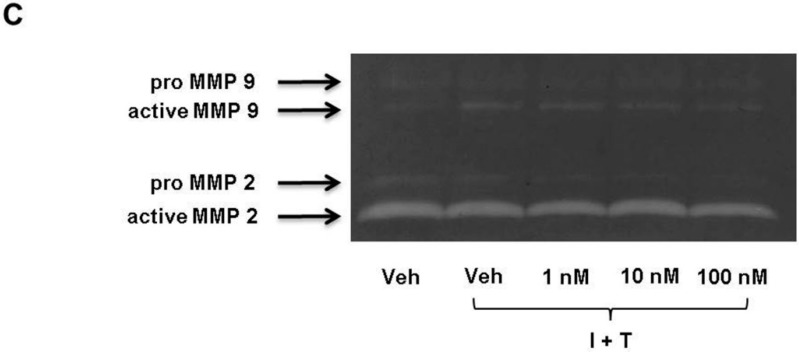
Secretion of matrix metalloproteinase (MMP)-2 (**A**) and MMP-9 (**B**) by rat brain microvascular endothelial cells after exposure to interferon γ and tumor necrosis factor α (I + T; 100 IU each) and treatment with FTY720-P (FTY-P; 1, 10 and 100 nM; *n* = 4) for 18 h compared with untreated cultures under inflammatory conditions (Vehicle I + T; *n* = 4) as well as with cultures in a homeostatic milieu (Vehicle; *n* = 4); (**C**) Representative experiment of zymography bands. ns, not significant; Veh, vehicle.

MMPs increase the permeability of the blood-brain barrier by attacking the extracellular matrix, basal lamina and TJs in endothelial cells, resulting in the final neuroinflammatory damage [19]. To examine whether FTY720-P alters MMP-2 or MMP-9 expression of endothelial cells, we performed a zymographic analysis of RBMEC supernatants 18 h after FTY720-P treatment. We detected no difference between FTY720-P- and vehicle-treated BMEC cultures regarding the amount of MMP-2 and MMP-9 (Figure 4A–C).

### 2.3. FTY720-P Reduces Occludin in RBMEC Cultures

According to the aforementioned experiments, FTY720-P did not alter BBB permeability in an RBMEC culture exposed to inflammatory conditions. This finding raised the question whether FTY720-P might even have the opposite effect, *i.e.*, might further destabilize the endothelial barrier function—an eventuality which is not necessarily depicted by measurements of the TEER. Therefore, we investigated the effect of FTY720-P on the TJ proteins occludin, claudin 5, and ZO-1 in RBMEC cultures which were exposed to inflammatory conditions for 18 h. Quantitative assessment of TJ proteins was performed using Western blot analysis followed by densitometric quantification; a qualitative determination of these proteins was enabled by immunocytochemistry.

**Figure 5:**
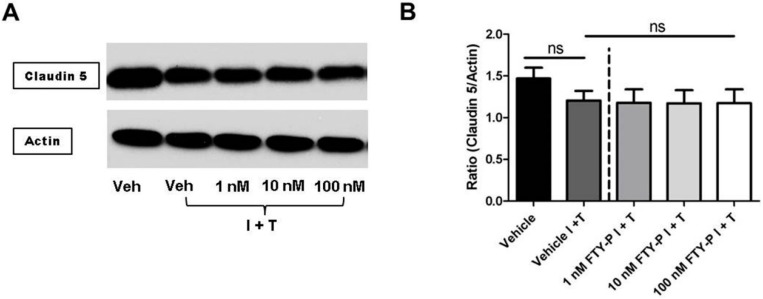

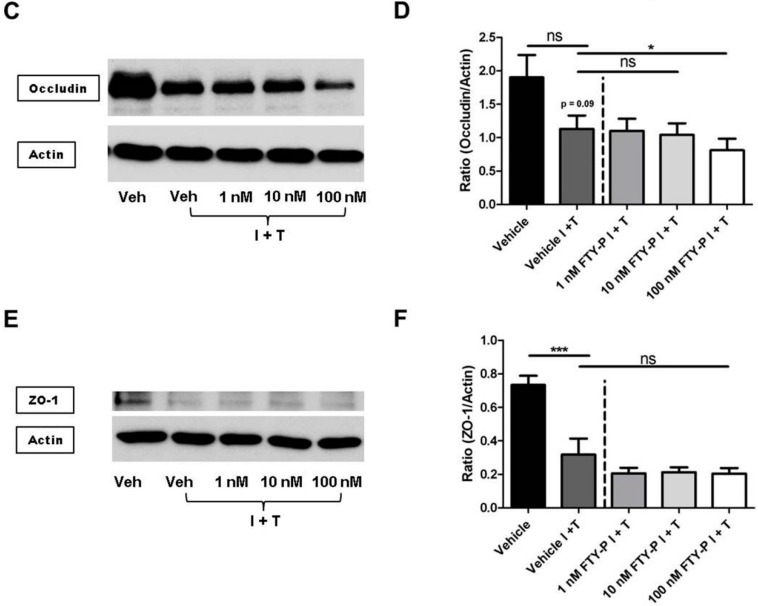
Effect of FTY720-P (FTY) on the amount of junctional protein of rat brain microvascular endothelial cell (RBMEC) cultures under inflammatory conditions. Western blot analysis and densitometric quantification of the amount of claudin 5 (**A**,**B**); occludin (**C**,**D**); and ZO-1 (**E**,**F**) proteins in RBMECs after exposure to interferon γ and tumor necrosis factor α (I + T; 100 IU each) and treatment with FTY-P (1, 10 and 100 nM; *n* = 4) for 18 h compared with untreated cultures under inflammatory conditions (Vehicle I + T; *n* = 4) as well as with cultures in a homeostatic milieu (Vehicle; *n* = 4). β-Actin was used as loading control. * *p* < 0.05; *** *p* < 0.001; ns, not significant; Veh, vehicle.

When FTY720-P was added to RBMEC cultures under inflammatory conditions, a dose-dependent and significant decrease of occludin was detected compared with non-treated cell cultures (occludin/actin 1.1 ± 0.2 *vs.* 0.8 ± 0.2; *p* < 0.05). In contrast, FTY720-P-treatment did not change the amount of claudin 5 in RBMEC cutlures when exposed to an inflammatory milieu (Figure 5, Figure S1). Additionally, ZO-1 was significantly decreased in RBMEC cultures after exposure to IFNγ and TNFα. However, FTY720-P-treatment did not alter the amount of ZO-1.

### 2.4. FTY720-P-Associated Decrease of Occludin Is Not Mediated via the erk1/2 Pathway

**Figure 6:**
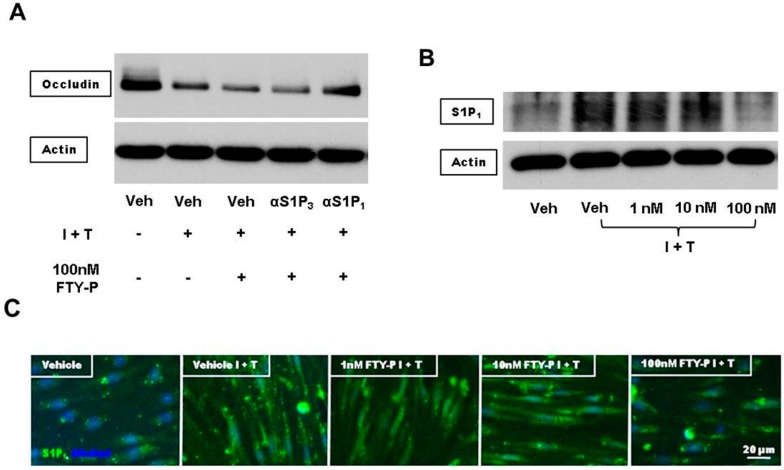
(**A**) Representative Western blot analysis of the amount of occludin and β-actin proteins in RBMECs either exposed to interferon γ and tumor necrosis factor α (I + T; 100 IU each) or cultured in physiological medium (Veh) after incubation with FTY720-P (100 nM) in the presence or absence of a specific S1P_1_ or S1P_3_-receptor antagonist (αS1P_3_, αS1P_1_; 10 μM each) for 18 h; (**B**) Representative Western blot analysis of the amount of S1P_1_ and β-actin proteins RBMECs either exposed to interferon γ and tumor necrosis factor α (I + T; 100 IU each) or cultured in physiological medium after incubation with FTY720-P (1, 10, and 100 nM) for 18 h; (**C**) Representative immunofluorescence staining against Hoechst (blue) and S1P_1_ (green) in RBMECs.

FTY720-P is a functional antagonist of all S1P receptors except S1P_2_ [20]. Therefore we next examined whether RBMEC expresses these receptors under inflammatory conditions. In contrast to the S1P_1_ and S1P_3_-receptor expression, we observed a sdownregulation of S1P_4_ and S1P_5_-receptors under inflammatory conditions (supplementary Figure S2). Thus, S1P_4_ and S1P_5_-receptors seem to play no crucial role in the regulation of the BBB integrity under inflammatory conditions and therefore no further investigations on these two receptors were performed. To analyze whether FTY720 impacts S1P_1_ or S1P_3_-receptor signaling we used both, specific S1P_1_ and S1P_3_-receptor antagonists. Our Western blot analyses yielded only a link between S1P_1_ signaling and the reduction of occludin (Figure 6A).

Downregulation of occludin is induced by activation of the extracellular-signal-regulated kinase (erk1/2) pathway leading to an increase in endothelial permeability [21]. erk1/2 phosphorylation (*i.e.*, activation) is mediated by high-affinity antagonism of S1P_1_, including a ligand-induced receptor internalization [22]. We next assessed the effect of FTY720-P on the protein amount of S1P_1_. There was no change in the amount of S1P_1_ protein when cell cultures were treated with FTY720-P doses of 1 or 10 nM. However, treatment with 100 nM FTY720-P revealed a clear reduction in this amount (Figure 6B,C).

**Figure 7:**
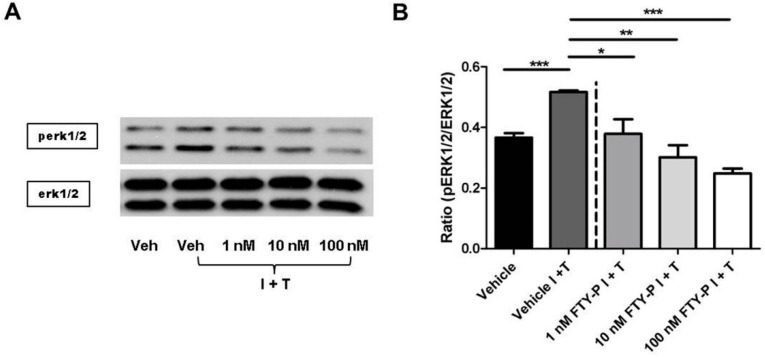
Effect of FTY720-P (FTY-P) on erk1/2 signaling in rat brain microvascular endothelial cell (RBMEC) cultures exposed to inflammatory conditions and incubated with FTY720-P. (**A**,**B**) Western blot analyses and densitometric quantification of the amount of erk1/2 and perk1/2 proteins in RBMECs treated for 90 min with FTY-P (1, 10, and 100 nM; *n* = 3) and exposed to interferon γ and tumor necrosis factor α (I + T; 100 IU each) compared with untreated cultures under inflammatory conditions (Vehicle I + T; *n* = 3) as well as to cultures in homeostatic milieu (Vehicle; *n* = 3). * *p* < 0.05; ** *p* < 0.01; *** *p* < 0.001; Veh, vehicle.

Ninety minutes after exposing RBMEC culture to IFNγ and TNFα, phosphorylation of erk1/2 decreased by adding FTY720-P to the cell cultures at doses of 1, 10, and 100 nM in a dose-dependent manner (Figure 7).

## 3. Discussion

In the present study, the role of FTY720-P on endothelial permeability has been examined in RBMEC cultures exposed to inflammatory conditions. As a main finding, FTY720-P at different dosages did not decrease endothelial permeability in RBMECs after exposure to IFNγ and TNFα. This is in contrast to recently published studies which revealed a protective effect of FTY720-P on BBB leakage in cell cultures and in an animal model [14,23]. However, following FTY720-P treatment in MS patients, macular edema was a prominent adverse event reported in two large randomized controlled trials, the TRANSFORMS [24] and FREEDOMS [12] trial. When comparing different studies of FTY720-P in MS [12,24] and renal transplant recipients [25,26], the incidence of macular edema was dose-dependent. Interestingly, whereas the ocular toxicity was dose-dependent in these studies, the therapeutic effect was not [27]. The pathogenesis of FTY720-P-associated macular edema is unspecified. There is some evidence that FTY720-P involves also the degradation of the S1P_1_ receptor and thus plays a role in regulating vascular permeability [28,29], providing a possible explanation of FTY720-P-associated macular edema.

Furthermore, we found a downregulation of the TJ protein occludin in RBMEC cultures exposed to inflammatory conditions and treated with FTY720-P. The S1P_1_ receptor is involved in the intercellular adhesion of endothelial cells via TJs, AJs and gap junctions [28,30]. ZO-1, a TJ-associated protein, forms a multiprotein complex with occludin [31,32] and thus is suggested to regulate occludin. S1P alters the distribution of ZO-1 protein via S1P_1_ which results in an enhanced endothelial barrier function [30,33]. FTY720-P is a functional antagonist to S1P_1_ and, as a consequence, might reduce occludin in RBMECs.

Redistribution of ZO-1 protein is mediated via the S1P_1_/G_i_/Akt/Rac pathway [30], which finally results in erk1/2 phosphorylation. Activation of erk1/2 has been shown to downregulate occludin [21]. In the present study, assessment of erk1/2 revealed a dose dependent decrease in phosphorylation but also a decrease of occludin after FTY-720-P-treatment under inflammatory conditions. Thus, we hypothesize that regulation of occludin is also mediated by mechanisms that overcome the S1P_1_/G_i_/Akt/Rac pathway. Increased MMP-9 activity promotes the disruption of occludin and ZO-1 in cerebral endothelial cells leading to the disruption of BBB integrity [34]. Additionally, MMP-9 is also able to degrade occludin in retinal epithelial cells [35]. However, in this study, the amount of MMP-9 and ZO-1 in BMECs did not change when FTY720-P was added to the medium, making this pathway unlikely. Alternatively, a reduction of occludin might be promoted via caveolin-1, a major structural component of membrane microdomains thought to be functionally complexed with TJs [36]. Caveolin-1 siRNA delivered to BMEC monolayers reduced the caveolin-1 protein level as well as occludin and ZO-1. In this context, occludin exhibited a dissociation from the cytoskeletal framework [37]. Downregulation of ZO-1/occludin is also associated with a decreased expression of connexin 43 (Cx43) [38], a gap junction protein, which is required to maintain endothelial barrier function [39]. Notably, the interaction of Cx43 and ZO-1/occludin is linked to S1P_1_ modulation [40].

In summary, FTY720-P did not contribute to a decreased leakage of endothelial barrier function of RBMECs after exposure to inflammatory conditions, which is in line with other studies [22,41,42]. In contrast, we found downregulation of the TJ protein occludin, which rather contributes to a further destabilization of the endothelial barrier. This observation might be due to S1P_1_-mediated signaling since this receptor intervenes in the regulation of endothelial permeability, providing a mechanistic basis for FTY720-P-associated disruption of barrier function. Conversely, S1P_1_ activation via S1P enhances endothelial barrier integrity through its action both on the cell cytoskeleton and on intercellular junctions [43]. Therefore, S1P_1_ has a dual role in immune and vascular cell biology. Depending on whether S1P or FTY720-P interacts with this receptor, S1P_1_-mediated signaling either enhances endothelial barrier function or inhibits lymphocyte egression from lymphoid tissues and increases vascular permeability.

## 4. Materials and Methods

### 4.1. Materials

FTY720-P was obtained from Biomol (Hamburg, Germany). FTY720-P was dissolved in the vehicle solution composed of DMSO/HCl (stock concentration: 50 mM) and diluted with methanol to 1 mM FTY720-P [44]. IFNγ was purchased from Miltenyi (Bergisch Gladbach, Germany). TNFα was obtained from Peprotech (Hamburg, Germany). Selective sphingosine-1-phosphate receptor S1P_1_ (W146) and S1P_3_ (TY 52156) antagonists were obtained from Tocris (Wiesbaden-Nordenstadt, Germany). Rabbit anti-caspase 3, anti-erk1/2, and anti-Phospho-erk1/2 antibodies were acquired from Cell Signaling (Leiden, The Netherlands). Rabbit anti-S1P_1_ was obtained from Santa Cruz (Heidelberg, Germany). Rabbit anti-vWF, and anti-occludin were purchased from Abcam (Cambridge, UK). Mouse anti-claudin 5 and rabbit anti-ZO-1 antibodies were obtained from Invitrogen (Darmstadt, Germany). Mouse anti-CD31 and rabbit anti-vWF antibodies were obtained from Invitrogen. Mouse anti-occludin antibody was acquired from Becton Dickinson (Heidelberg, Germany). All other reagents were purchased from Sigma–Aldrich (St. Louis, MO, USA).

### 4.2. Preparation and Cultivation of RBMECs

RBMECs were isolated and cultured as previously described for murine brain microvessel endothelial cells [45]. Briefly, brains of eight-week-old rats were homogenized and stepwise digested by two proteases, followed by further purification steps. RBMECs were either planted on collagen type IV/fibronectin-coated transwell polyester membrane inserts with 0.4-μm pores (Corning, Life Science, Kaiserslautern, Germany) or in 24-well plates (Nunc, Darmstadt, Germany). The cell cultures were maintained in serum-containing culture medium at 37 **°**C with a humidified atmosphere of 5% CO_2_/95% air, for five days. All experiments were conducted in serum-free medium.

### 4.3. Immunocytochemistry

RBMECs were cultured on collagen IV/fibronectin-coated glass coverslips or Transwell Polyester Membrane inserts with 0.4-μm pores. Cells were fixed with methanol for 10 min at −20 °C and stained with mouse anti-CD31, rabbit anti-vWF, rabbit anti-occludin, rabbit anti-S1P_1_, or mouse anti-claudin 5 according to standard protocols. RBMECs were imaged with a Nikon Eclipse 50i microscope equipped with a CCD camera (Nikon, Tokyo, Japan).

### 4.4. Western Blot Assays

RBMECs were cultured in 24-well plates until they reached confluency. After cell lysis, the amount of protein was determined using BCA protein assay. Denatured protein (1–3 μg) was electrophoresed and transferred to a nitrocellulose membrane. Membranes were blocked for 1 h and incubated with either an anti-erk1/2 antibody, anti-phosphoerk1/2, anti-S1P_1_, anti-caspase 3, anti-claudin 5, antu-ZO-1, or an anti-occludin antibody at 4 °C overnight. Thereafter, the membranes were incubated with a horseradish peroxidase-conjugated anti-rabbit or anti-mouse immunoglobulin G antibody (Dianova, Hamburg, Germany) at room temperature for 1 h. Immunoblots were detected using ECLplus (PerkinElmer, Waltham, MA, USA) and a Kodak X-OMAT 5000 RA developer (Kodak, Rochester, NY, USA). In order to control protein loading and transfer, membranes were incubated with a β-actin monoclonal antibody. Bands were quantified by densitometric analysis using ImageJ Analysis Software 1.45s [46] and normalized to the actin band.

### 4.5. Measurement of TEER

The electrical resistance across the RBMEC layers was measured using an automated long-term monitoring resistance meter (CellZcope, San Francisco, CA, USA). TEER was assessed on endothelial cell monolayers cultured on collagen IV/fibronectin-coated Transwell Pore Polyester Membrane inserts with 0.4-μm pores (Corning).

### 4.6. Zymography

The amount of secreted MMP-2 and MMP-9 in BMEC supernatants was detected by zymography as recently reported [47]. Briefly, BMEC supernatants were collected and mixed with equal volumes of sample buffer (20% glycin, 4% SDS, 2 mM EDTA, 0.01% bromophenol blue, 125 mM Tris–HCl pH 6.8). Next, samples were loaded on a 10% SDS-PAGE containing 0.1% gelatin. After electrophoresis, the gels were treated twice with 2.7% Triton X-100 solution for 30 min and thereafter were incubated in developing buffer (50 mM Tris, 200 mM NaCl, 5 mM CaCl_2_, 0.02% Brij-35, 40 mM Tris–HCl pH 7.5) at 37 °C overnight. Gels were stained with 0.25% (*w*/*v*) Coomassie brilliant blue in 25% isopropanol/10% acetic acid, and bleached with 50% methanol and 10% acetic acid until bands with diminished staining appeared. Images of gels were captured by EPSON Perfection V500 scanner (Epson, Nagano, Japan) and subsequently analyzed with ImageJ Analysis Software 1.45s National Institutes of Health, Bethesda, MD, USA.

### 4.7. Quantitative Real-Time PCR

Quantitative real-time PCR analysis was performed according to standard procedures. Relative gene expression levels of S1P_1_ (*EDG1*; assay ID: Rn02758712_s1; Applied Biosystems, Frankfurt, Germany), S1P_3_ (*EDG3*; assay ID: Rn02758880_s1, Applied Biosystems), S1P_4_ (*EDG4*; assay ID: Rn01408095_s1, Applied Biosystems), and S1P_5_ (*EDG5*; assay ID: Rn00572952_s1, Applied Biosystems) were quantified with fluorescent TaqMan technology. *Gapdh* (TaqMan Predeveloped Assay Reagents for gene expression, part number: 4351371, Applied Biosystems) was used as an endogenous control.

### 4.8. Statistical Analysis

All results are presented as mean ± SEM. To test for significant differences between multiple groups, one-way analysis of variance was used, with post hoc Bonferroni adjustment for *p*-values. Statistical analysis comparing two groups (Vehicle *vs.* Vehicle I + T) was performed using two-tailed Student *t* test. *p-*values <0.05 were considered significant with * *p* < 0.05; ** *p* < 0.01; *** *p* < 0.001.

## Supplementary Materials

Supplementary materials can be found at http://www.mdpi.com/1422-0067/16/12/26177/s1.
