# Supplementary material for: Fingolimod (FTY720-P) Does Not Stabilize the Blood–Brain Barrier under Inflammatory Conditions in an in Vitro Model
Source: Int J Mol Sci. 2015 Dec 10;16(12):29454–66. doi: 10.3390/ijms161226177 (PMC4691120; doi:10.3390/ijms161226177)
Supplement: Supplementary file 1 [file ijms-16-26177-s001.pdf]

Supplementary Materials: Fingolimod (FTY720-P) Does Not Stabilize the Blood–Brain Barrier under Inflammatory Conditions in an *in Vitro* Model

Michael K. Schuhmann, Stefan Bittner, Sven G. Meuth, Christoph Kleinschnitz and Felix Fluri

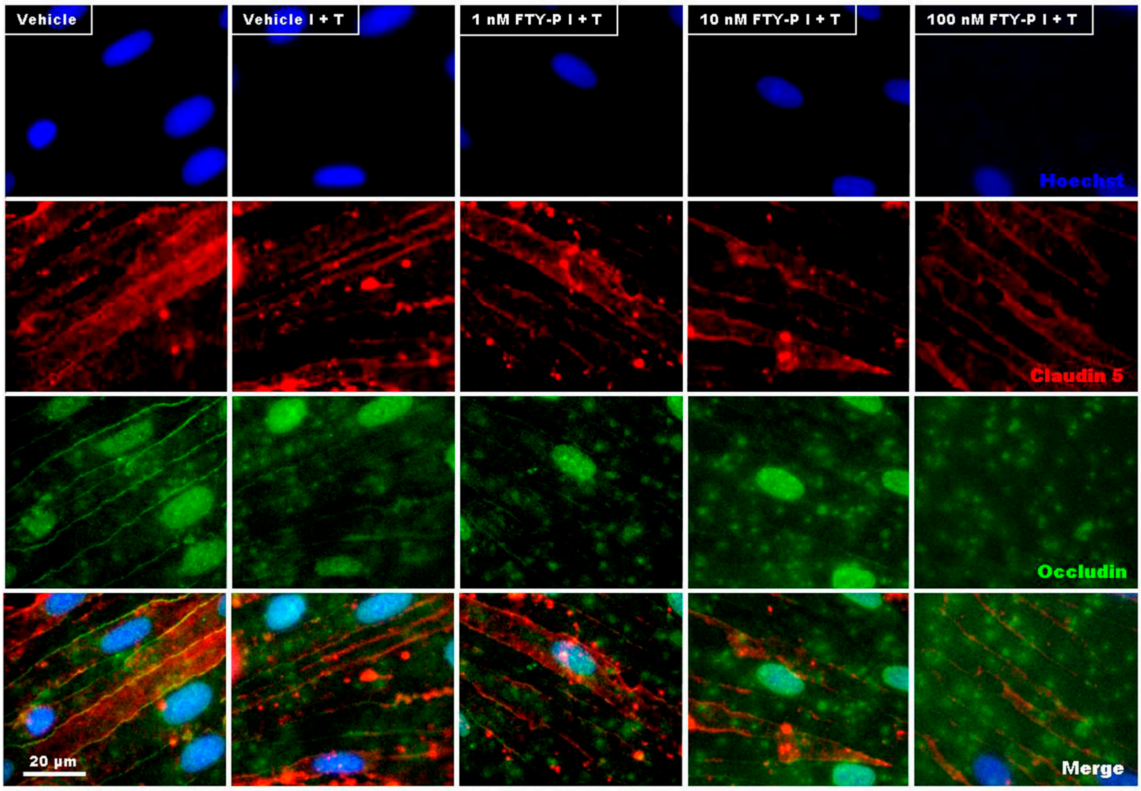

Figure S1. RBMEC immunofluorescence staining against Hoechst (blue), claudin 5 (red), and occludin (green) 18 h after exposure to interferon γ and tumor necrosis factor α (I + T; 100 IU each) and FTY720-P treatment.

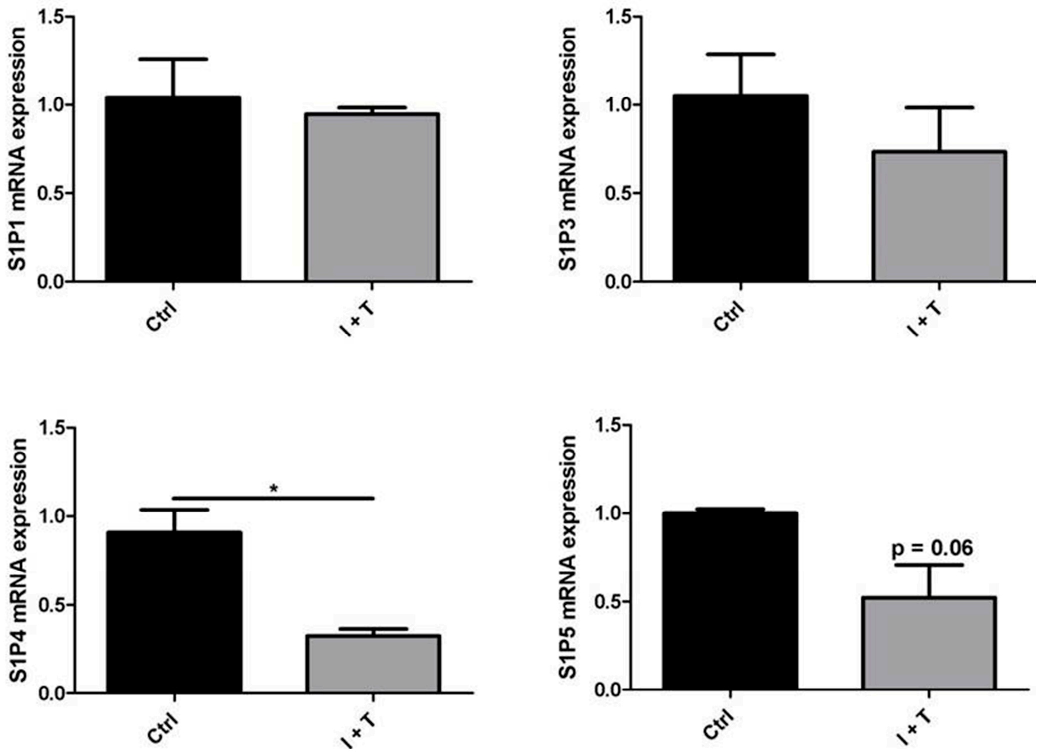

Figure S2. RT-PCR analysis of $S1P_1$, $S1P_3$, $S1P_4$, and $S1P_5$ –receptor (n = 3) mRNA expression (normalized to Ctrl) in rat brain microvascular endothelial cells (RBMECs) exposed to interferon γ and tumor necrosis factor α (I + T; 100 IU each) or cultured in physiological medium (Ctrl) for 6 h. * $p < 0.05$.
